# Recent progress in drug development for fibrodysplasia ossificans progressiva

**DOI:** 10.1007/s11010-022-04446-9

**Published:** 2022-05-10

**Authors:** Xinmiao Meng, Haotian Wang, Jijun Hao

**Affiliations:** 1 College of Arts and Sciences, Cornell University, Ithaca, NY 14850 USA; 2 College of Arts and Sciences, University of Pennsylvania, Philadelphia, PA 191041 USA; 3 College of Veterinary Medicine, Western University of Health Sciences, Pomona, CA 91766 USA

**Keywords:** Fibrodysplasia ossificans progressiva, ACVR1, BMP, TGF-β, ALK2, Heterotopic ossification

## Abstract

Fibrodysplasia Ossificans Progressiva (FOP) is a rare genetic disease caused by heterozygous missense mutations in Activin A receptor type I which is also known as Activin-like kinase 2 (ALK2), a type I receptor of Bone Morphogenetic Proteins(BMP). Patients with FOP usually undergo episodic flare-ups and the heterotopic ossification in soft and connective tissues. Molecular mechanism study indicates that Activin A, the ligand which normally transduces Transforming Growth Factor Beta signaling, abnormally activates BMP signaling through ALK2 mutants in FOP, leading to heterotopic bone formation. To date, effective therapies to FOP are unavailable. However, significant advances have recently been made in the development of FOP drugs. In this article, we review the recent advances in understanding the FOP mechanism and drug development, with a focus on the small-molecular and antibody drugs currently in the clinical trials for FOP treatment.

## Introduction

**Fig. 1:**
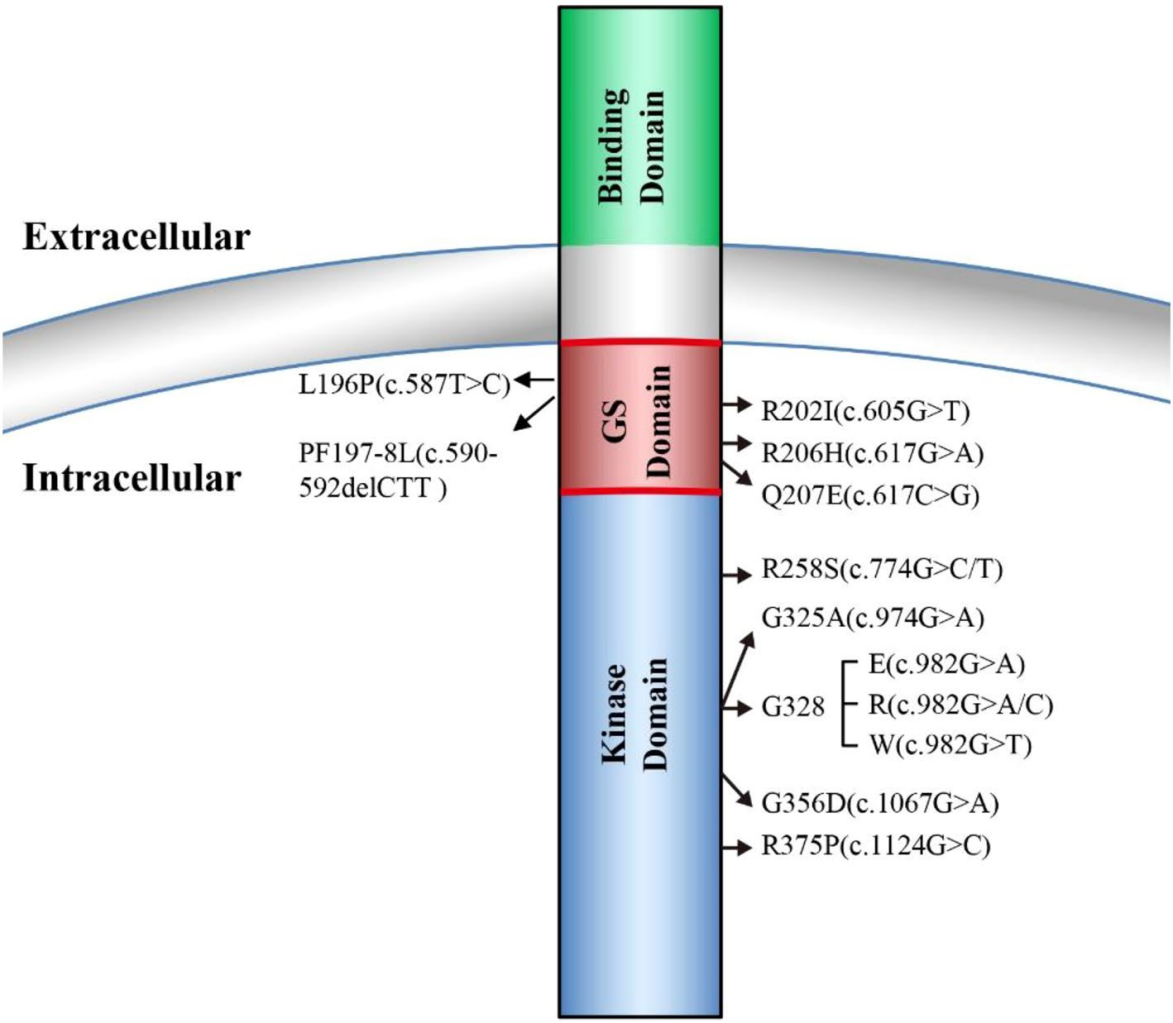
FOP causative mutations in ALK2 (ACVR1). ALK2 consists of ligand-binding domain, transmembrane domain, GS-rich domain, and serine/threonine kinase domain. All the identified FOP causative mutations are located either in either GS-rich domain or the serine/threonine kinase domain

FOP is a rare human genetic disorder in which ectopic bone formation occurs in connective tissue such as tendons, ligaments, and skeletal muscles throughout the body, leading to progressive loss of mobility, chronic pain, and eventual premature death mainly due to cardiorespiratory failure [1]. A worldwide prevalence of FOP is approximately one in two million population without ethnic, racial, or geographic predisposition [2]. One main symptom of FOP is a malformation of big toes at birth which also serves as an early diagnostic hallmark for FOP [2, 3]. In 2006, the first heterozygous missense causative mutation of FOP (617G>A; R206H) was reported in the gene-encoding ACVR1 [4]. Since then, additional new heterozygous missense causative mutations in ACVR1 have been reported, and further studies indicated that ACVR1^R206H^ mutation occurs in approximately 97% of FOP patients [5, 6] (Fig. 1). ACVR1, also known as ALK2, is a type I receptor of BMP signaling essential for normal skeleton formation and embryonic patterning [7, 8]. For a more complete view of FOP etiology, clinical characteristics, diagnosis, and management, we refer the readers to the excellent reviews in these topics [2, 3, 9].

Early mechanistic studies showed that FOP ALK2 mutants result in leaky BMP signaling in a basal condition and hyper-responsiveness upon BMP ligand stimulation [10–17]. However, recent findings have confirmed that activin A, the ligand which normally transduces TGF-β signaling, abnormally activates BMP signaling through FOP-mutated ALK2 [18–21]. This abnormal activin A-induced BMP signaling is thought to trigger heterotopic ossification of connective tissues [22]. To date, although effective therapies for FOP are unavailable, significant advances have been achieved in the development of potential FOP drugs, resulting in several promising therapies currently in clinical trials [23]. In this article, we review the recent progress in FOP mechanism studies and drug development, with a focus on the small-molecular and antibody drugs in the clinical trials for FOP treatment.

### BMP signaling and FOP

**Fig. 2:**
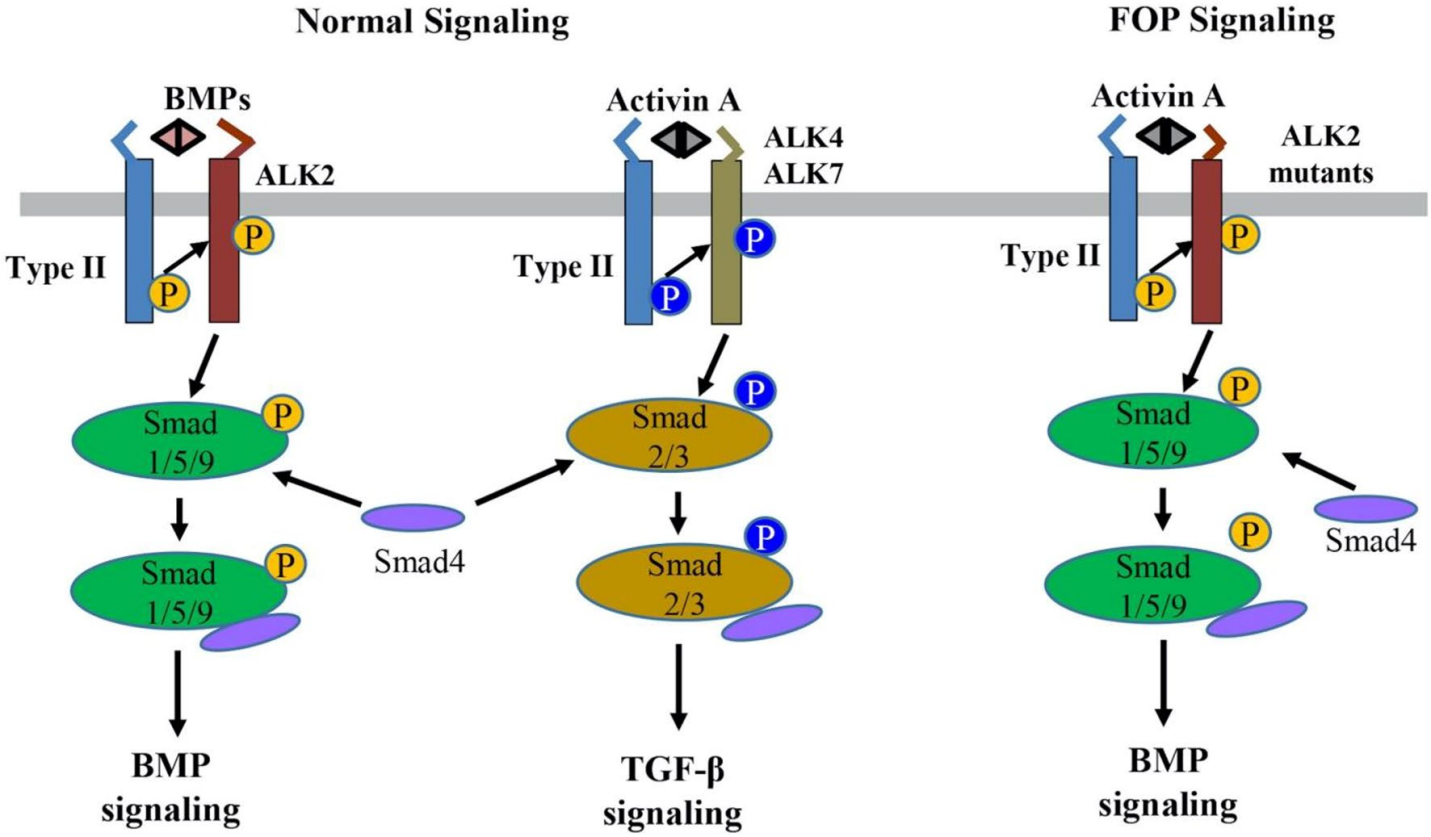
The normal BMP/TGF-β signaling pathways and abnormal activin A-induced BMP signaling through the ALK2 mutants in FOP. BMP or activin A ligands assemble and bind to a heterotetramer complex consisting of a type II receptor homodimer and a type I receptor homodimer (e.g., ALK2 for BMP and ALK4/7 for activin A). The type II receptor phosphorylates the type I receptor, which subsequently phosphorylates Smads (Smad1/5/9 for BMPs and Smad2/3 for activin A) to transduce normal BMP and TGF-β signaling, respectively. In contrast, in FOP, activin A can abnormally cross-signal BMP signaling through the ALK2 mutants

BMPs are secreted multi-functional growth factors, and they belong to the TGF-β super family. BMPs consist of more than 20 family members which play central roles in regulating cellular morphogenesis, differentiation, proliferation, and apoptosis during embryogenesis and adult homeostasis [24]. The BMPs signal transduction is mainly mediated through the canonic Smads-dependent pathway in which BMPs first bind to a heterotetrametric complex consisting of a type II receptor homodimer and a type I receptor homodimer (Fig. 2). Then the type II receptors phosphorylate and activate the type I receptors, which in turn phosphorate Smad1/5/9 (also known as Smad1/5/8). The phosphorylated Smad1/5/9 subsequently form a complex with Smad4, which then translocates into the nucleus where it binds to BMP response elements and activates transcription of BMPs target genes [24, 25].

Four type I receptors, ALK1, ALK2, ALK3, and ALK6, are able to mediate BMP signaling and malfunctions of these four types I receptors are involved in many diseases including cancer [26, 27]. In FOP, the most common mutation R206H is located at the intracellular glycine-serine-rich (GS) domain of ALK2, where FKBP12 protein (also known as FKBP1A) binds to ALK2 to prevent ALK2 activation in the absence of BMP ligands [12, 15, 16]. ALK2^R206H^ has been shown to induce basal leaky BMP signaling in the absence of BMP ligands and hyper-responsiveness upon BMP ligand stimulation that was initially thought to result in the ectopic endochondral ossification in FOP [15–17, 28]. Later, additional FOP mutations have been identified in both GS domain and kinase domain of ALK2, which are associated with the disease onset ages and the extent of heterotopic ossification [5, 10, 29–32].

Nevertheless, recent findings have proved that activin A, a ligand which normally transduces TGF-β signaling, abnormally activates BMP signaling in FOP [18–21]. In normal physiological conditions, BMPs utilize ALK1/ALK2/ALK3/ALK6 as the type I receptors to activate Smad1/5/9-dependent BMP signaling, while activin A signals through ALK4/ALK7 as the type I receptors for Smad2/3-dependent TGF-β signaling and activin A does not transduce Smad1/5/9-dependent BMP signaling [33] (Fig. 2). However, recent multiple studies have demonstrated that activin A can activate Smad1/5/9-dependent BMP signaling in cells expressing ALK2^R206H^ in vitro and induced heterotopic ossification in a conditional knock-in mouse model of FOP in vivo [18–21, 34, 35]. In addition, this heterotopic ossification in the FOP mouse model can be blocked by the activin A-specific antibodies supporting that activin A cross-signal BMP pathway via mutated FOP ALK2 receptors [18–21]. Advances in understanding of the FOP molecular mechanism have led to significant progress in FOP drug development.

### Recent drug development for FOP

Based on the molecular mechanism underlying FOP, multiple potential therapeutic targets have been selected for drug development to treat the disease.

### Targeting ALK2

Since FOP is caused by the missense mutations of ALK2, ALK2 has been long thought as a potential therapeutic target for FOP and significant efforts have been made to develop ALK2 inhibitors.

**Fig. 3:**
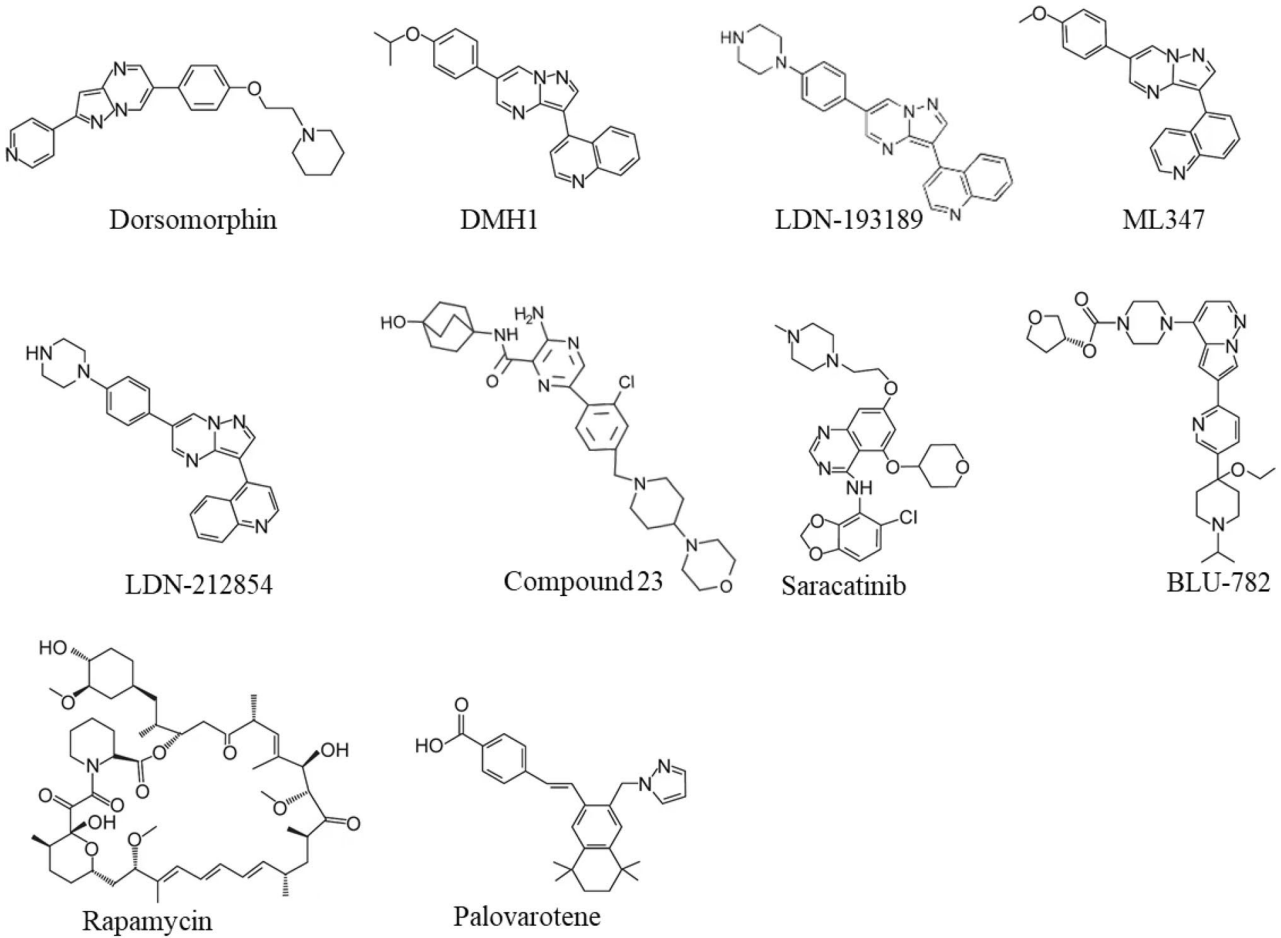
Chemical Structures of Small-Molecular Inhibitors of ALK2, Rapamycin, and Palovarotene

Dorsomorphin, the first ALK2 chemical inhibitor, was identified from an in vivo screening of BMP inhibitors using zebrafish embryos [36] (Fig. 3). Unfortunately, Dorsomorphin displays notable off-targets against serval other kinases including Vascular Endothelial Growth Factor Receptor 2 (VEGFR2), ALK5, AMP-activated kinase (AMPK) and platelet-derived growth factor receptor β (PDGFRβ) [37], raising concerns about its clinical safety [37, 38]. To develop more selective ALK2 inhibitors, we and colleagues have synthesized 63 Dorsomorphin analogs and identified DMH1 from those analogs by using zebrafish embryo screening [37]. In contrast to Dorsomorphin, DMH1 is more selective to ALK2, and it does not exhibit detectable activities against the closely related kinases such as VEGFR2, ALK5, AMPK, and PDGFRβ [37]. Meanwhile, another ALK2 inhibitor, LDN-193189, was developed, and it shows better potency and selectivity than Dorsomorphin [39] (Fig. 3). Nevertheless, both DMH1 and LDN-193189 cannot well distinguish ALK2 from other BMP type I receptors (ALK1/3/6) which are essential for development and homeostasis [40–43]. Therefore, developing better ALK2 inhibitor is critical for FOP treatment with minimum side effects. Further investigations discovered more selective ALK2 inhibitors, ML347 and LDN-212854 with negligible inhibitory activities for all other kinases except ALK1 [44, 45] (Fig. 3). Very recently, Ullrich et al. reported a new potent and selective ALK2 inhibitor, compound 23, which displays excellent biochemical and cellular potency, selectivity, and a favorable in vitro profiles for absorption, distribution, metabolism, and excretion [46]. However, none of the above selective ALK2 inhibitors have moved into clinical trials.

Recently, Williams et al. screened over 220 small-molecular kinase inhibitors which have either been approved previously by FDA or in clinical trials [47]. They identified a potent and selective ALK2 inhibitor, Saracatinib (also known as AZD0530), an orally bioavailable drug developed by AstraZeneca for the treatment of ovarian adenocarcinoma [47, 48] (Fig. 3). Since Saracatinib effectively blocks heterotopic ossification in preclinical FOP models and displays excellent pharmacokinetic parameters and safety, Phase II clinical trial of Saracatinib for FOP was recently initiated in August 2020 (NCT04307953) [49, 50] (Table 1). Another selective ALK2 inhibitor, INCB000928 that was originally developed to treat anemia as an iron homeostasis modulator, is now being evaluated for the efficacy and tolerability in the treatment of FOP in the phase II clinical trial (NCT05090891) [51, 52] (Table 1). Other than small-molecular ALK2 inhibitors, an anti-ALK2 monoclonal antibody, DS-6016a, was developed as well by Daiichi Sankyo and Saitama Medical University in Japan. The Phase I clinical trial of DS-6016a to assess its safety, tolerability, and pharmacokinetics in healthy participants is ongoing, and the study results have not been released to date (NCT04818398) [53] (Table 1).

**Table 1:** Recent clinical trials for FOP (by November 2021).

Drug name	Clinical phase	Target	NCT/UMIN number
Saracatinib	Phase II	ACVR1	NCT04307953
INCB000928	Phase II	ACVR1	NCT05090891
DS-6016a	Phase I	ACVR1	NCT04818398
BLU-782 (IPN60130)	Phase I	ACVR1	NCT03858075
REGN2477 (Garetosmab)	Phase II	Activin A	NCT03188666
Rapamycin	Phase II/III	mTORC1	UMIN000028429
Palovarotene	Phase III	Nuclear RARγ	NCT03312634 NCT05027802

*NCT* the national clinical trial, *UMIN* university hospital medical information network

Nevertheless, these ALK2-targeting potential drugs indiscriminately target both wild-type ALK2 and FOP-mutated ALK2, leading to inhibition of important physiologic BMP signaling essential for normal cellular and tissue function. To overcome this challenge, Blueprint Medicines, Inc. developed a small molecule called BLU-782 (also known as IPN60130), which selectively targets the FOP-mutated ALK2 with minimal interference to the wild-type ALK2 [54] (Fig. 3). The Phase I clinical trial BLU-782 in healthy volunteers to establish its safety of the investigational drug was recently completed (NCT03858075), and the result showed that BLU-782 is well tolerated with approximately 24 h of half-life and displays excellent properties of pharmacokinetics and pharmacodynamics [55, 56] (Table 1).

### Targeting activin A

Activin A normally mediates TGF-β signaling by using Activin Receptors type IIA or IIB (ActR-IIA/ActR-IIB) as type II receptors and ALK4/7 as type I receptors followed by the downstream-phosphorylated Smad2/3 as intracellular signal transducers (Fig. 2). However, recent studies have confirmed that activin A abnormally activates BMP-Smad1/5/9 signaling through mutant ALK2 in FOP [18–21, 34, 35]. Given this interesting discovery, activin A has become a promising therapeutic target for FOP treatment. REGN2477 (also known as Garetosmab), a human anti-activin A-neutralizing antibody, was examined in the FOP mouse model, and the result showed that REGN2477 effectively inhibited heterotopic ossification [19]. The Phase I clinical trial of REGN2477 was completed, and the result demonstrated that REGN2477 displays great safety, tolerability, and pharmacokinetics [57]. Recently its Phase II clinical trial was initiated with a plan to administer 10 mg/kg REGN2477 intravenously every 4 weeks to FOP patients (NCT03188666) [58]. As activin A also plays important roles in multiple biological functions such as ovarian follicle maturation, spermatogenesis, steroidogenesis, muscle growth, immunity, inflammation, neuronal differentiation, and bone remodeling [59–64], the potential side effects of REGN2477 for activin A inhibition must be carefully monitored in FOP patients (Table 1).

### Targeting other associated transcriptional effectors

It is believed that activin A induces chondrogenesis via BMP signaling in FOP by differentiating connective tissue progenitor cells into chondrocytes and osteoblasts prior to eventual formation of heterotopic bones in soft tissues [34, 65]. Thus, inhibition of chronogenesis may be a good strategy to prevent heterotopic ossification in FOP.

#### Rapamycin

Rapamycin (also known as Sirolimus) is an immunosuppressive drug used to prevent transplant rejection and lymphangioleiomyomatosis, and it has been recently identified as a potential drug for the treatment of FOP (Fig. 3). In a high-throughput screening by using FOP patient-derived induced pluripotent stem cells (FOP-iPSCs) to identify signaling pathways involved in activin A-induced chondrogenesis, Hino et al. found that the mammalian target of rapamycin (mTOR) signaling is critical in enhanced chondrogenesis initiated by activin A and heterotopic ossification in FOP [66]. They further showed that Rapamycin attenuated heterotopic ossification in both FOP-ALK2^R206H^ conditional transgenic mice and the mice with activin A-triggered heterotopic ossification derived from FOP-iPSCs [66]. Given the promising preclinical studies and its proved safety profile, Phase II/III clinical trials of Rapamycin for randomized, placebo-controlled studies and subsequent open-label extension studies were initiated at Kyoto University Hospital in Japan (UMIN000028429), and the outcomes of this trial has not been publicly released (Table 1). Nevertheless, a case report recently showed that Rapamycin did not show clear benefits to heterotopic ossification reduction in two young patients with classic FOP-ALK2^R206H^ mutation at the administrated dose [67].

#### Palovarotene

Retinoid signaling mediated by nuclear retinoic acid receptors (RAR) plays a critical biological role in chondrogenesis and normal skeleton formation and retinoic acid signaling agonists could effectively block chondrogenesis and subsequent heterotopic ossification in FOP [68–71]. In 2011, Shimono et al. showed that palovarotene (also known as R667), a specific agonist of the retinoic acid signaling by targeting nuclear retinoic acid receptor-γ (RARγ) with well characterized safety profile, inhibited heterotopic ossification in a transgenic mouse model expressing ALK2^Q207D^ mutation [72] (Fig. 3). Later, Chakkalakal et al. examined palovarotene in a knock-in mouse model carrying the classic FOP-ALK2^R206H^ mutation and demonstrated that palovarotene effectively blocks trauma-induced and spontaneous heterotopic ossification without comprising limb mobility and growth [73]. Importantly, palovarotene maintained joint, limb, and body motion, providing clear evidence for its encompassing therapeutic potential as a treatment for FOP [73]. In 2014, Clementia Pharmaceuticals initiated a double-blinded, placebo-controlled Phase II clinical trial to evaluate whether palovarotene prevents heterotopic ossification during and following a flare-up in FOP patients (NCT02190747). The trial was completed in 2016, and the result shows that palovarotene reduces the percentage of FOP patients developing heterotopic ossification, the time to remission and patient-reported pain associated with the flare-up area [74]. Currently, the Phase III clinical trial of palovarotene in FOP patients is in progress (NCT03312634). In addition, the rollover Phase III study was launched in November 2021 to further evaluate the safety and efficacy of palovarotene in adult and pediatric participants with FOP who have previously received palovarotene treatment (NCT05027802) [75] (Table 1).

## Conclusion

In recent years, significant progresses have been made in understanding the molecular mechanism underlying FOP and developing FOP therapies. The discovery of causative mutations in ALK2 has made it a promising druggable target for FOP. Numerous small-molecular inhibitors and antibodies targeting ALK2 have been developed. Among them, Saracatinib, DS-6016a, and BLU-782 are currently in FOP clinical trials. In addition, as activin A abnormally transduces BMP signaling in FOP, REGN2477 antibody-targeting activin A has been studied for the treatment of FOP, and its efficacy is currently under evaluation in a Phase II clinical trial. Moreover, potential drugs targeting transcriptional effectors associated with the early heterotopic ossification have also shown promise in the treatment of FOP, and their efficacies are being evaluated in clinical trials. For instance, a Phase II clinical trial has showed that RARγ agonist Palovarotene effectively reduces the percentage of FOP patients developing heterotopic ossification and the time to remission (NCT02190747) [74]. Additionally, Rapamycin was shown to attenuate heterotopic ossification in FOP mouse models [66], and a Phase II clinical trial for Rapamycin is currently ongoing. In summary, rapid, and exciting advances have been made in our understating of FOP mechanism and drug development. Several potential drugs are currently under clinical trials to treat FOP at multiple targets, which allows more effective combinatorial pharmacological management for FOP. Nevertheless, as physiological BMP signaling is critical to homeostasis and indiscriminately blocking BMP signaling to treat FOP may raise some concerns, therapeutic agents like BLU-782 that selectively targets only the mutant ALK2 with minimal interference to the wild-type ALK2 may represent an excellent strategy for FOP treatment in the future.

## Data Availability

Not applicable.
